# CAWS administration increases the expression of interferon γ and complement factors that lead to severe vasculitis in DBA/2 mice

**DOI:** 10.1186/1471-2172-14-44

**Published:** 2013-09-24

**Authors:** Noriko Nagi-Miura, Daisuke Okuzaki, Kosuke Torigata, Minami A Sakurai, Akihiko Ito, Naohito Ohno, Hiroshi Nojima

**Affiliations:** 1 Laboratory for Immunopharmacology of Microbial Products, School of Pharmacy, Tokyo University of Pharmacy and Life Sciences, 1432-1 Horinouchi, Hachioji, Tokyo 192-0329, Japan; 2 Department of Molecular Genetics and, Research Institute for Microbial Diseases, Osaka University, 3–1 Yamadaoka, Suita, Osaka 565-0871, Japan; 3 DNA-chip Development Center for Infectious Diseases, Research Institute for Microbial Diseases, Osaka University, 3–1 Yamadaoka, Suita, Osaka 565-0871, Japan; 4 Department of Pathology, Faculty of Medicine, Kinki University, 377-2 Ohno-Higashi, Osaka-Sayama, Osaka 589-8511, Japan

**Keywords:** CAWS, DNA microarray, DBA/2, Kawasaki disease, Complement factors, Ifng

## Abstract

**Background:**

*Candida albicans* water-soluble fraction (CAWS), a mannoprotein-β-glucan complex obtained from the culture supernatant of *C. albicans* NBRC1385, causes CAWS-mediated vasculitis (CAWS-vasculitis) in B6 and DBA/2 mice with mild and lethal symptoms, respectively. Why CAWS is lethal only in DBA/2 mice remains unknown.

**Results:**

We performed DNA microarray analyses using mRNA obtained from peripheral blood mononuclear cells (PBMCs) of B6 and DBA/2 mice and compared their respective transcriptomes. We found that the mRNA levels of interferon-γ (Ifng) and several genes that regulate the complement system, such as C3, C4, Cfb, Cfh, and Fcna, were increased dramatically only in DBA/2 mice at 4 and 8 weeks after CAWS administration. The dramatic increase was confirmed by quantitative real-time polymerase chain reactions (qRT-PCR). Moreover, mRNA levels of immune-related genes, such as Irf1, Irf7, Irf9, Cebpb, Ccl4, Itgam, Icam1, and IL-12rb1, whose expression levels are known to be increased by Ifng, were also increased, but only in DBA/2 mice. By contrast, the mRNA level of Dectin-2, the critical receptor for the α-mannans of CAWS, was increased slightly and similarly in both B6 and DBA/2 mice after CAWS administration.

**Conclusions:**

Taken together, our results suggest that CAWS administration induces Dectin-2 mediated CAWS-vasculitis in both B6 and DBA/2 mice and the expression of Ifng, but only in DBA/2 mice, which led to increased expression of C3, C4, Cfb, Cfh, and Fcna and an associated increase in lethality in these mice. This model may contribute to our understanding of the pathogenesis of severe human vasculitis.

## Background

Vasculitis is characterized by the localized infiltration of inflammatory cells into the intravascular wall and its surroundings, and is accompanied by denaturation and necrosis, leading to potentially life-threatening conditions [1]. The *Candida albicans* water-soluble fraction (CAWS), a mannoprotein-β-glucan complex obtained from the culture supernatant of *C. albicans* NBRC1385, exhibits vasculitis-inducing activity in mice (CAWS-vasculitis), which acts as a trigger for the induction of vasculitis in the coronary artery [2-4]. Indeed, CAWS has strong induction potency for murine vasculitis and induces lethal toxicity in certain mouse strains [5,6]. Unlike polypeptides derived from *Candida albicans*[7], however, CAWS does not display superantigen-like activities because the part of the CAWS complex that elicits physiological activity is the polysaccharide chain. Dectin-2, a C-type lectin expressed by dendritic cells (DCs) and macrophages, functions as the critical DC receptor for α-mannans of CAWS and plays a pivotal role in host defense against *C. albicans* by inducing Th17 cell differentiation [8]. A subtype of CAWS with a higher ratio of β-mannosyl linkages, named CAWS727, failed to cause severe vasculitis in DBA/2 mice but inhibited the inflammatory response via a competitive association with α-mannan specific lectins, including dectin-2 [9]: this result indirectly demonstrated dectin-2 involvenment in the development CAWS mediated vasculitis. Intraperitoneal injection of CAWS induced coronary arteritis in both C57BL/B6 (B6 hereafter) and DBA/2 mice [10,11]. However, only DBA/2 mice exhibited severe necrotizing coronary arteritis and aortitis leading to nearly 100% mortality within weeks from stenosis in the left ventricular outflow tract and severe inflammatory changes of the aortic valve with fibrinoid necrosis [12]. Although Dectin-2 may be essential for CAWS-vasculitis in both B6 and DBA/2 mice, it remains elusive as to why CAWS-vasculitis is fatal to DBA/2 mice, but not to B6 mice.

The complement system, comprised of more than 30 serum and cellular proteins, plays a major role in innate immune defenses against infectious agents [13]. Complement activation appears to be involved in the pathogenesis of systemic autoimmune diseases [14]. The alternative pathway involving C3 convertase (C3bBb), a potent enzymatic protein complex capable of rapidly converting C3 into powerful effector fragments (C3a and C3b), amplifies the initial response, inducing a variety of effector functions [15]. In acute coronary syndrome (an inflammatory disease), the complement cascade is activated and the C3 and C4 concentration ratio (C3/C4 ratio) in serum is a readily available marker of recurrent cardiovascular events [16]. Complement activation produces the complement C5 (C5), a precursor to the effector molecules, C5a and C5b; C5a acts as a trigger for the cellular immune response and C5b acts as an initiator of the formation of the membrane attack complex [17]. C5a mediates inflammatory responses [18] through the C5a receptor 1 (C5aR); however, C5a also exerts an anti-inflammatory effect [19], putatively through its second receptor, the C5a-like receptor 2 (C5L2), which may act either as a decoy receptor or by forming the C5aR/C5L2/β-arrestin complex, depending on the cell type, species and disease context [20]. Notably, DBA/2 mice are defective in C5, though it remains unknown how this C5 deficiency may contribute to the fatal effect of CAWS.

Ifng, the only member of the type II class of interferons, is a soluble homodimer with potent proinflammatory properties that plays critical roles in innate and adaptive immunity against viral and intracellular bacterial infections. Ifng-induced inflammation is involved in aging and aging-associated medical and psychiatric disorders [21]. Notably, Ifng level was increased in splenocyte culture supernatants following stimulation with lipopolysaccharide [11], by a Gram-negative bacterial cell wall component or with CAWS [10,22]. Thus, it appears that complement factors and Ifng are somehow involved in CAWS-vasculitis; however, no direct data to confirm this have been reported.

In the present study, we report the results of DNA microarray analysis of mRNAs from PBMCs comparing differences in gene expression patterns in B6 and DBA/2 mice. We found that mRNA levels of Ifng and complement factors such as C3, C4, Cfb, Cfh, and Fcna were conspicuously augmented only in DBA/2 mice at 4 and 8 weeks after CAWS administration. Moreover, mRNA levels of immune-related genes, such as Irf1, Irf7, Irf9, Cebpb, Ccl4, Itgam, Icam1, and IL-12rb1, whose expression levels are known to be augmented by Ifng, were also increased only in DBA/2 mice. Based on these results, we propose a molecular model to explain how CAWS treatment induces fatal vasculitis in DBA/2 mice.

## Results

### Induction of arteritis by injection of CAWS into DBA/2 mice

**Figure 1:**
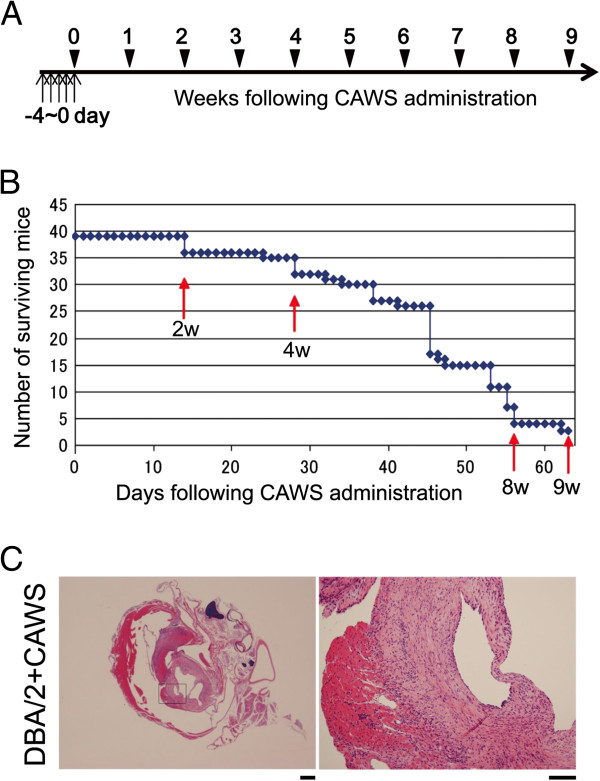
**Survival curve and histopathological analysis. (A)** Schedule of CAWS administration. Vertical arrows indicate that CAWS was administered *i.p.* (0 or 1 mg/mouse) for 5 consecutive days to each B6 or DBA/2 mouse. **(B)** The surviving number of DBA/2 mice following CAWS administration is shown, and confirms that CAWS administration is as effective (toxic) as in our previous report [12]. Red vertical arrows indicate the time points (2 w, 4 w, 8 w and 9 w) at which three B6 mice and three DBA/2 mice were sacrificed for mRNA purification. **(C)** Histopathological analysis was performed on aortic roots and coronary arteries collected from DBA/2 mice at 6 w following CAWS administration stained with H&E. Enlarged views of the region indicated by rectangles in the left panels are shown in the right panels. Bar = 500 μm for left panels and 100 μm for right panels.

To induce arteritis in B6 (control) and DBA/2 mice, we performed intraperitoneal injection (*i.p.)* of CAWS (0 or 1 mg/mouse) for 5 consecutive days as reported previously [11]; we will call this process as CAWS administration, hereafter, and set this timing (right after the 5^th^ injection of CAWS) as 0 week (0 w). Although the number of surviving mice at 2 w, 4 w, 8 w, and 9 w following CAWS administration was remarkably reduced in DBA/2 mice (Figure 1A), similar to our previous report [11], few B6 mice died during this period (data not shown), which is also similar to our previous report [11]. At 2 w, 4 w, 8 w, and 9 w after CAWS administration, the surviving mice were sacrificed and mRNA isolated from PBMCs. Three mice were sacrificed at each time point (the number of sacrificed mice is included in Figure 1B). Tissue sections that were obtained from paraffin-embedded hearts fixed with 10% neutral formalin and stained with hematoxylin and eosin (H&E) revealed vasculitis in the aorta and coronary artery of DBA/2 mice, with fibrotic thickening of the arterial media and adventitia and infiltration of inflammatory cells (Figure 1C). In tissue sections obtained from the hearts of mice at 2 w, 4 w, 8 w, and 9 w following CAWS administration, we observed infiltration of inflammatory cells in the aortic media and adventitia of DBA/2 mice, a histological finding typical of aortitis, as early as 2 w following CAWS administration, and this was observed up to the final 9 w time point (right panels in Additional file 1: Figure S1). B6 mice also showed infiltration of inflammatory cells in the aortic media and adventitia, although the onset of aortitis in B6 mice occurred later than in DBA/2 mice, not appearing until 4–6 w following CAWS administration (left panels in Additional file 1: Figure S1). Since these results were similar to those of our previous report [10,11], we concluded that the mice used for DNA microarray analysis in this study responded to CAWS in a similar manner to those described in our previous report.

### CAWS enhanced expression of complement system genes only in DBA/2 mice

To identify genes whose expression levels were up- or downregulated in the PBMCs of DBA/2 mice, but not in B6 mice, following CAWS treatment, we examined the transcriptome profiles of mRNA prepared from DBA/2 and B6 PBMCs at 2 w, 4 w, 8 w, or 9 w following CAWS administration using Agilent’s Whole Mouse Genome Microarray (G4122F). When the relative intensities of the signals at 2 w, 4 w, 8 w, or 9 w were compared with those at 0 w following CAWS administration, the genome-wide expression profiles were found to be distinct between these two mouse strains (Additional file 1: Figure S2A). Heat map representation of the signal intensities revealed that a number of genes were dramatically up- or downregulated in DBA/2 mice compared to B6 mice (Additional file 1: Figure S2B).

**Figure 2:**
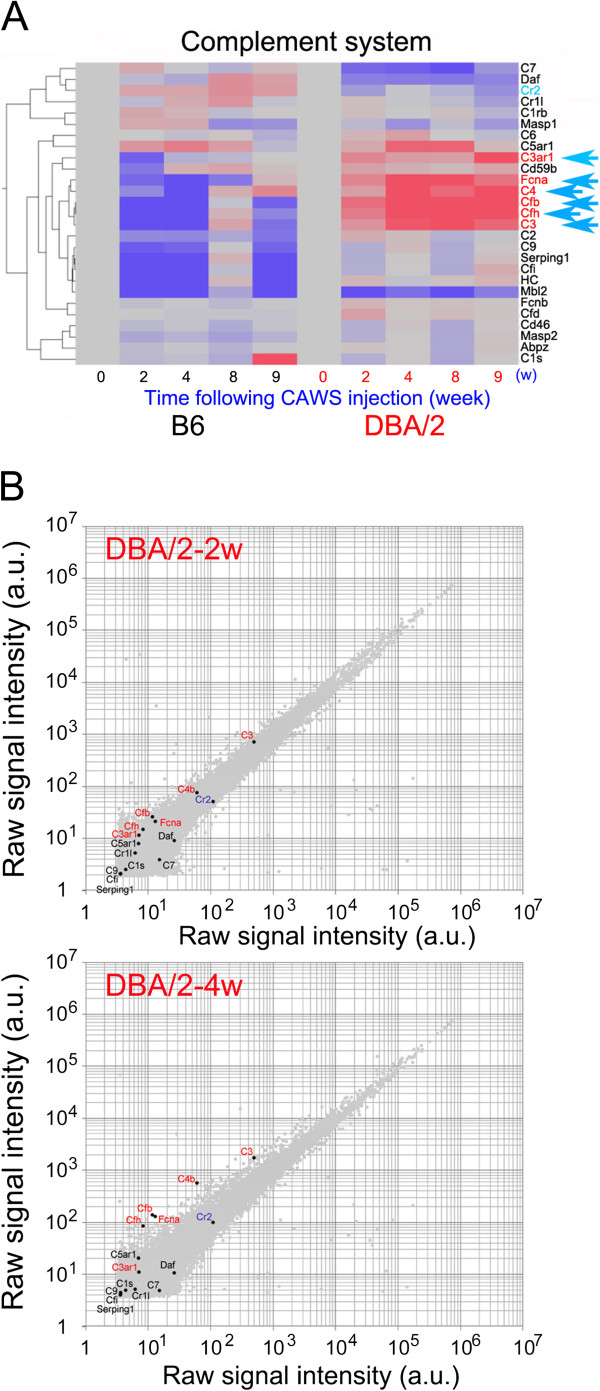
**Expression profiling of microarray data for complement system genes following administration of CAWS to B6 and DBA/2 mice. (A)** Mosaic tile representation of genes involved in the complement system. B6 and DBA/2 samples were clustered using a hierarchical clustering program (Spearman) to identify gene-to-gene relationships. Intensity gradients indicate the mean value of the expression level (log2 ratio): blue (down-regulation) and crimson (upregulation) are shown compared to the average value at week 0 (0 w) after CAWS administration (gray). C3ar1, Fcna, C4, Cfb, Cfh, and C3 genes are highlighted by turquoise arrows. **(B)** Scatter plots of the log of signal intensity at 2 w (top panel) or 4 w (bottom panel) versus 0 w of CAWS administration in DBA/2 mice. Data are plotted over the entire signal intensity range to demonstrate that these expression arrays provide a high-resolution platform. C3ar1, Fcna, C4, Cfb, Cfh, and C3 genes are highlighted in red font.

The most notable genes that showed enhanced expression following CAWS treatment in DBA/2 mice belong to the “complement system”, in particular the genes encoding C3, C4, complement factor b (Cfb or BF), complement factor h (Cfh or HF1), and ficolin-A (Fcna) (indicated by turquoise arrows in Figure 2A and Additional file 1: Figure S2B). The complement 3a receptor 1 (C3ar1) gene showed modestly enhanced expression at 2 w, 4 w, and 8 w following CAWS administration and conspicuously enhanced expression at 9 w (turquoise arrow in Figure 2A). C5ar1 gene also showed enhanced expression at 4 w and 8 w, but B6 mice also showed enhanced expression at this timing, albeit less dramatically. Expression of complement C9 (C9), Serping1 (*Serpin* peptidase inhibitor, clade *G1*), complement factor i (Cfi), and hemolytic complement (HC) was extremely low in B6 mice at 2 w, 4 w and 9 w, but their expression was detectable in DBA/2 mice, displaying a comparatively enhanced ratio in DBA/2 mice compared with B6 mice. By contrast, complement C7 (C7), decay accelerating factor (DAF or CD59), and complement receptor 2 (Cr2) showed decreased expression following CAWS treatment in DBA/2 mice, although these genes were weakly expressed in B6 (Figure 2A).

To determine whether changes in the expression levels of these genes is physiologically significant, a scatter plot was used to analyze the distribution of the expression levels of many genes at a glance. Indeed, C3, C4, Cfb, Cfh, Fcna, and Cr2 showed expression levels high enough to conduct physiologically significant comparison (>10 raw signal intensity) in DBA/2 mice at 2 w or 4 w following CAWS administration (Figure 2B). By contrast, complement genes that showed very low expression levels compared to above described activated complement genes in both B6 and DBA/2 mice were not deemed significant (Figure 2B). C3ar1 mRNA levels were low at 2 w or 4 w and its expression level was enhanced only at 9 w following CAWS administration. Thus, we conclude that the enhanced mRNA levels of C3, C4, Cfb, Cfh, and Fcna observed after CAWS administration in DBA/2 mice are significant.

### The alternative complement pathway is activated in DBA/2 mice following CAWS administration

**Figure 3:**
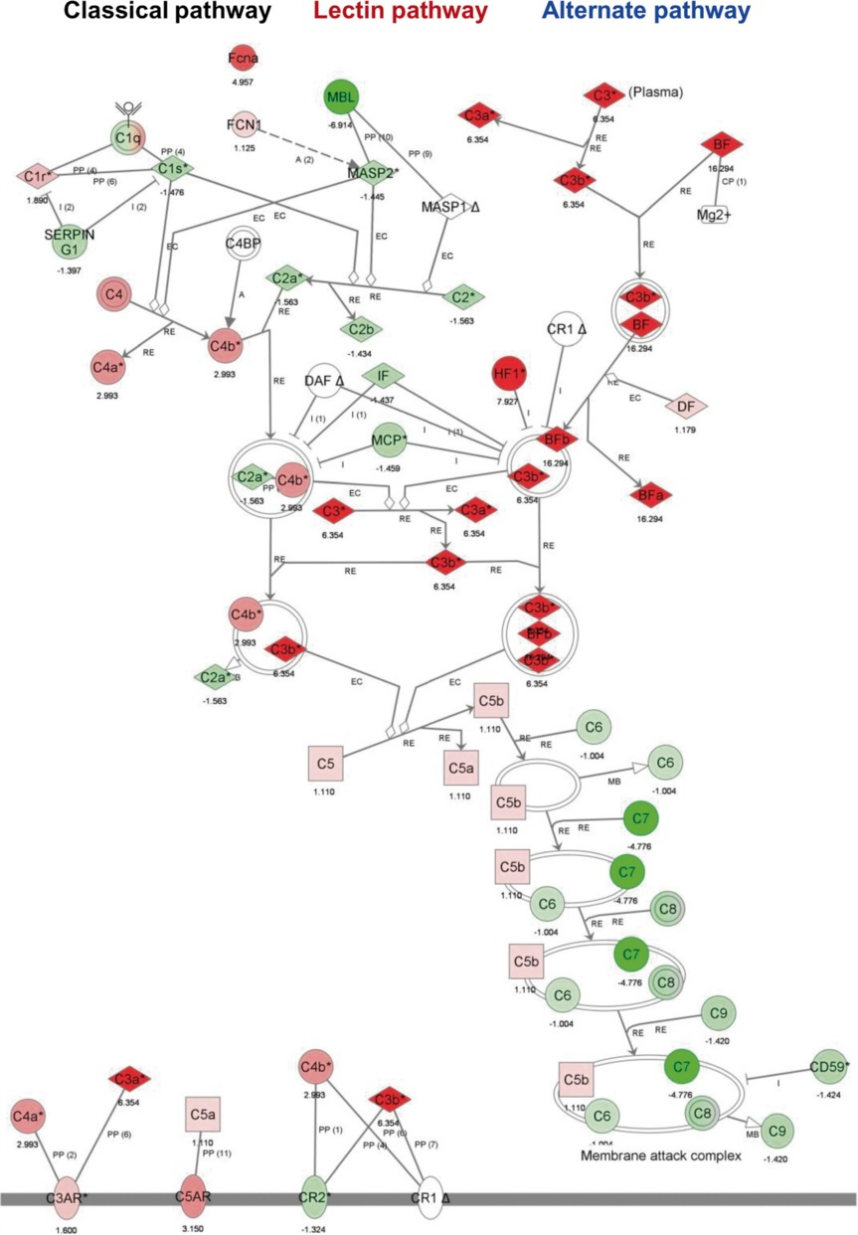
**Analysis of the transcriptome data.** The data were obtained from PBMCs following administration of CAWS to B6 and DBA/2 mice using Ingenuity Pathway Analysis software. This analysis suggested that among the three pathways of the complement system, the alternative pathway appears to be conspicuously activated. A network pathway is a graphical representation of the molecular relationships between molecules. Molecules are represented as nodes, and the biological relationship between two nodes is represented as an edge (line). All edges are supported by at least one reference from the literature, a textbook, or canonical information stored in the Ingenuity Knowledge Base. The green and red bars associated with each gene symbol indicate up- (red) and downregulated (green) mRNA expression at 2 w, 4 w, 8 w, or 9 w after CAWS administration in B6 (left; green bars) and DBA/2 mice (right; red bars). Upregulated and downregualted levels were measured as fold changes with respect to levels at 0 w.

We next performed an ontology search using Ingenuity Pathways Analysis (Ingenuity Systems, http://www.ingenuity.com). This powerful query system facilitates the rapid visualization of relationships between sets of relevant genes to predict the putative physiological significance of these genes. We found that alternative complement pathway genes, including C3, Cfb (=BF), and Cfh (=HF1), appear to play major roles in activation of the complement system (Figure 3). By contrast, classical pathway genes, including complement C1 (C1s and C1r) and Serping G1 (but not C4), and lectin pathway genes including mannose-binding lectin (Mbl) appear to play less significant roles because they showed low levels of expression even in DBA/2 mice (Figure 3). The low mRNA level of C7 measured in DBA/2 mice suggests that downstream regulation of the complement system is not important in the induction of vasculitis in the aortas or coronary arteries of DBA/2 mice.

**Figure 4:**
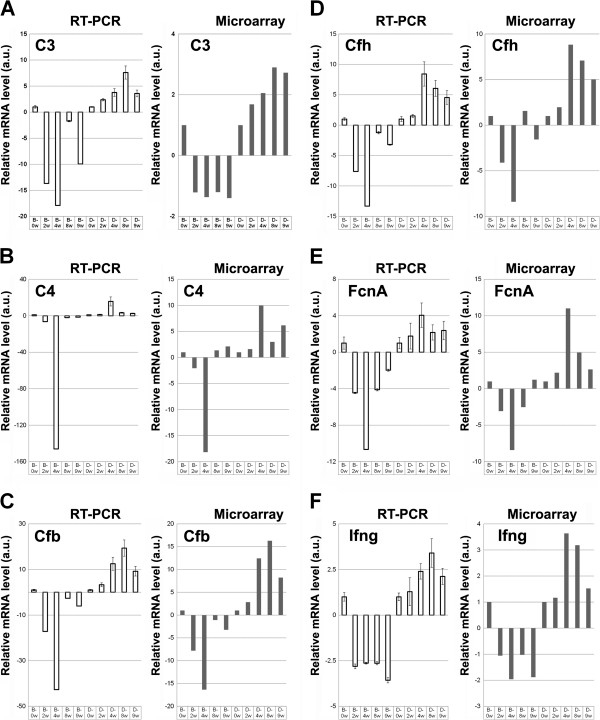
**Comparison of the mRNA expression levels.** The mRNA expression levels of C3 **(A)**, C4 **(B)**, Cfb **(C)**, Cfh **(D)**, FcnA **(E)**, and Ifng **(F)** were as assessed by qRT-PCR and DNA microarray of PBMCs obtained from B6 and DBA/2 mice at 2 w, 4 w, 8 w, and 9 w following CAWS administration. The vertical axis indicates the mRNA level (arbitrary unit: a.u.) relative to that at 0 w, which was fixed at 1.0 a.u.

To confirm that the microarray signals accurately reflected mRNA levels, expression profiling using quantitative reverse transcription-polymerase chain reaction (qRT-PCR) analysis was performed for C3, C4, Cfb, Cfh, and FcnA genes using the same RNA samples used for the microarray analysis. Indeed, all of the genes analyzed by qRT-PCR showed a similar pattern of mRNA distribution following CAWS administration; namely, they showed increased mRNA levels of the denoted genes in DBA/2 mice compared with B6 mice (Figure 4), confirming the accuracy of the microarray analysis data.

### CAWS induced upregulated expression of interferon-related genes only in DBA/2 mice

**Figure 5:**
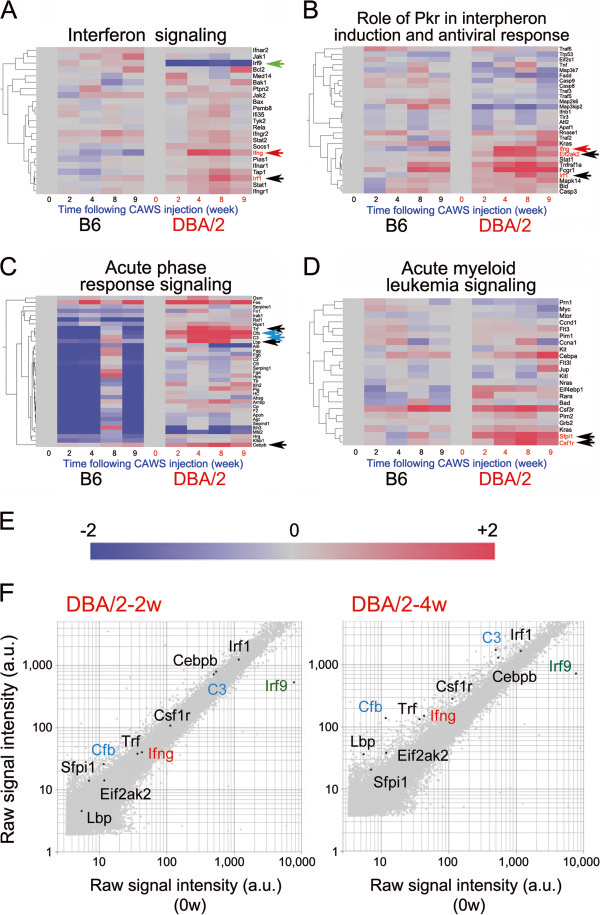
**Expression profiling of the microarray data for genes involved in interferon-related signal transduction pathways following administration of CAWS to B6 and DBA/2 mice.** Mosaic tiles and hierarchical clustering of microarray data are shown for the genes involved in interferon signaling **(A)**, the role of protein kinase R (Pkr) in interferon induction and antiviral response **(B)**, acute phase response signaling **(C)**, and acute myeloid leukemia signaling **(D)**. B6 and DBA/2 samples were clustered using a hierarchical clustering program (Spearman) to discover gene-to-gene relationships. Irf1, Eif2ak2, Trl, Lbp, Cebpb, Sfpil1, and Csf1r are highlighted by black arrows, Cfb and C3 by turquoise, Ifng by red and Irf9 by green arrows. **(E)** Intensity gradients indicate the mean value of the expression level (log2 ratio): blue (down-regulation) and crimson (upregulation) are shown compared to the average value at 0 w after CAWS administration (gray). **(F)** Scatter plots of the highlighted genes in log of signal intensity at 2 w (left panel) or 4 w (right panel) versus 0 w following CAWS administration in DBA/2 mice; data are plotted along the vertical and horizontal axes (arbitrary unit: a.u.), respectively. Highlighted genes are Ifng (red font), C3 (turquoise font), Cfb (turquoise font), and Irf9 (green font).

We next examined the expression of interferon-related genes because expression of the Ifng gene was enhanced following CAWS administration in DBA/2 but not in B6 mice, as revealed by both DNA microarray and qRT-PCR analyses (Figure 4F and Figure 5A). Notably, Ifng was the only interferon-related gene that exhibited upregulated DBA/2-specific expression following CAWS administration (Figure 5A): moreover, mRNA expression of Ifng was more conspicuously upregulated than that of other interferon gene subtypes in DBA/2 mice (Additional file 1: Figure S3). In the “interferon signaling pathway”, interferon regulatory factor-1 (Irf1) also showed increased expression, although less significantly than interferon itself, in DBA/2 mice at 4 w and 8 w (black arrow in Figure 5A). Notably, the mRNA level of Irf9 was decreased at 2 w, and this depressed level persisted throughout the experiment (green arrow in Figure 5A). This may explain why many of the interferon-stimulated genes were not activated (Figure 5A); the Irf9/Isgf3g complex binds to the interferon-stimulated response element to activate the transcription of interferon-stimulated genes [23].

In the pathway identified as the “Role of Prk in interferon induction and antiviral response”, mRNA levels of eukaryotic translation factor 2 alpha kinase 4 (Eif2aka) and Irf1 were enhanced (Figure 5B). Eif2aka is a heterotrimeric GTPase that controls the selection of correct start codons on mRNA, and thus regulates translation initiation by facilitating the preferential translation of selected transcripts in response to cellular stresses, in this case CAWS administration [24]. Our results suggest that DBA/2 mice are more sensitive to this type of stress than B6 mice.

In the “acute phase response signaling” pathway (Figure 5C), three genes other than C3 and Cfb were upregulated; CCAAT/enhancer-binding protein beta (Cebpb or C/EBPβ), IL-5, and lipopolysaccharide-binding protein (Lbp). Cebpb, a member of the C/EBP family of basic region-leucine zipper (bZIP) proteins, is largely expressed in macrophages and is important for the antibacterial activity of macrophages through binding to the regulatory regions of several acute phase and cytokine genes [25]. Enhanced expression of Cebpb following CAWS administration suggests that downstream target genes of Cebpb, including IL-4 (see below), may be widely activated. IL-5 exerts pleiotropic effects on various target cells, including B cells, eosinophils, and basophils, and overexpression of IL-5 *in vivo* significantly increases the number of eosinophils and B cells [26]. Lbp is involved in the acute phase immunologic response to Gram-negative bacterial infections through binding to bacterial lipopolysaccharide (LPS), thereby eliciting immune responses by presenting the LPS to the cell surface pattern recognition receptors CD14 and Tlr4 [27]. In this case, CAWS, rather than LPS, may have been recognized as an infectant.

In the “acute phase myeloid leukemia signaling” pathway (Figure 5D), two genes were upregulated: spleen focus-forming virus proviral integration 1 (Sfpi1 or PU.1) and colony stimulating factor 1 receptor (Csf1r). Sfpi1 is a transcription factor that plays a key role in hematopoietic cell fate decisions through mutual negative regulation that occurs between Sfpi1and GATA-1, a zinc finger transcription factor that is the central mediator of erythroid gene expression [28]. Csf1r is a single pass type I membrane protein that acts as a receptor tyrosine kinase for a cytokine called colony stimulating factor 1, which is a primary growth factor and monocyte/macrophage chemokine that regulates survival, proliferation and differentiation of macrophages and other mononuclear phagocytic lineage cells [29]. Enhanced expression of Csfr1 suggests the activation of macrophages following CAWS administration.

A scatter plot of Irf1, Irf9, C3, Cebpb, Csf1r, Trf, Ifng, Eif2aka, and Cfb expression showed expression levels high enough to conduct physiologically significant comparisons (>10 raw signal intensity) in DBA/2 mice at 2 w or 4 w following CAWS administration (Figure 2B), suggesting that their enhanced mRNA levels indicated by the heatmap (Figure 5E) are significant. By contrast, mRNA levels of Lbp and Sfpi1 may be too low for comparison between B6 and DBA/2 mice to be significantly different.

### Transcription factor genes showing upregulated expression only in DBA/2 mouse

**Figure 6:**
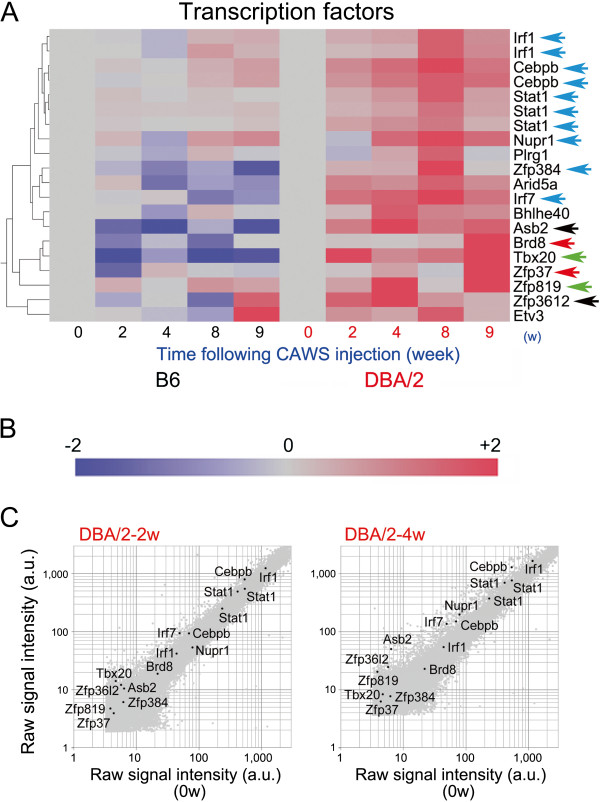
**Expression profiling of the microarray data for transcription factor genes following administration of CAWS to B6 and DBA/2 mice. (A)** Mosaic tiles and hierarchical clustering of microarray data are shown. B6 and DBA/2 samples were clustered using a hierarchical clustering program (Spearman) to identify gene-to-gene relationships. Genes upregulated predominantly in DBA/2 mice following administration of CAWS are highlighted by colored and black arrows. **(B)** Intensity gradients indicate the mean value of the expression level (log2 ratio): blue (down-regulation) and crimson (upregulation) are shown compared to the average value at 0 w after CAWS administration (gray). **(C)** Scatter plots of highlighted genes in the log of signal intensity at 2 w (left panel) or 4 w (right panel) versus 0 w following CAWS administration in DBA/2 mice; data are plotted along the vertical and horizontal axes (arbitrary unit: a.u.), respectively.

Some transcription factor genes also showed upregulated expression only in DBA/2 mice with mRNA levels peaking at 4 w (highlighted by black arrows in Figure 6), 8 w (turquoise arrows), 9 w (red arrows), and 2 w/9 w (green arrows). Irf1, Irf7, and Cebpb were discussed above. Stat1, a signal transducer and activator of transcription, plays a crucial role in the pathology of atherosclerosis resulting from the effects of interferon-γ produced by T cells. STAT1 also induces increased expression of several proinflammatory and pro-atherogenic mediators of signaling mediated by TLR4 [30]. Expression of Nupr1, a highly basic and loosely folded multifunctional protein, is induced in response to several cellular stresses [31] and provides protection against metabolic stress-mediated autophagic-associated cell death resulting from activation of the Nupr1-aurora kinase A pathway [32]. T-box 20 (Tbx20), a transcriptional regulator required for cardiac development, plays a dual role as both a transcriptional activator and a repressor, promoting or repressing distinct genetic programs within adult heart in response to cellular stress [33]. Increased expression with twin peaks observed at 2 w and 9 w suggests that CAWS injection altered regulation of Tbx20, thereby generating cardiac phenotypes (Figure 1C).

Zinc finger protein 384 (Zfp384), also called Nmp4 (nuclear matrix protein 4), is a nucleo-cytoplasmic shuttling transcription factor that regulates the expression of collagen and matrix metalloproteinases and represses bone formation induced by parathyroid hormone [34]. Ankyrin repeat and SOCS box-containing protein 2 (Asb2) interacts with mixed lineage leukemia (MLL) protein, a key epigenetic regulator of normal hematopoietic development, and overexpression of Asb2 degrades MLL to reduce its transactivation activity [35]. Zinc finger protein 3612 (Zfp3612), a member of the tristetraprolin family of tandem CCCH finger proteins that bind to AU-rich elements in the 3’-untranslated region of mRNAs, is a critical modulator of definitive hematopoiesis [36]. Zinc finger protein 819 (Zfp819), a member of the C2H2-zinc finger (C2H2-Znf) family of proteins, appears to function as a transcriptional and cell cycle/apoptosis regulator and play a role in the establishment and maintenance of pluripotency. Although these proteins play roles in several important cellular events such as hematopoiesis, these functions do not appear to be relevant to the pathogenesis of CAWS-induced vasculitis.

Scatter plot analysis indicated that mRNA levels of Irf1, Cebpb, Stat1, Nurp1, Irf7, and Brd8 were high enough to conduct physiologically significant comparison between B6 and DBA/2 mice according to their heatmaps (Figure 6B) at both 2 w and 4 w following CAWS administration (Figure 6C), suggesting that their superficially augmented expression is physiologically significant. By contrast, mRNA levels of Asb2, Zfp819, Zfp384, Zfp37, Zfp3612, and Tbx20 were too low to conclude that heatmap comparison of these genes between B6 and DBA/2 mice is physiologically significant.

Finally, CAWS administration resulted in a slight, but comparable increase in Dectin-2 mRNA levels in both B6 and DBA/2 mice (Additional file 1: Figure S7A). Moreover, scatter plot analysis indicated that the mRNA levels of Dectin-2 mRNA were lower than those of Dectin-1, a similar C-type lectin molecule (Additional file 1: Figure S7B).

## Discussion

In the present study, we used DNA microarray analysis to identify genes whose mRNA levels in PBMCs were upregulated in DBA/2 mice (with lethal vasculitis) but not in B6 mice (with non-lethal vasculitis) at 2 w, 4 w, 8 w, and 9 w following CAWS administration (Figure 1). We found that the mRNA levels of C3, C4, Cfb, and Cfh (Figure 2), which regulate the alternative pathway of the complement system (Figure 3), are conspicuously increased in DBA/2 mice at 4 w, 8 w, and 9 w following CAWS administration, whereas B6 mice showed decreased mRNA levels of these proteins, and this notable difference was confirmed by qRT-PCR (Figure 4). We also observed that mRNA levels of Ifng and interferon-related genes were augmented in the PBMCs of DBA/2 mice compared with those of B6 mice (Figure 4F and Figure 5A). Ifng expression is induced upon infection by pathogens such as viruses, bacteria, or parasites, suggesting that some components of CAWS, possibly the polysaccharide chains of α and β mannoproteins in the cell wall extract, are recognized as pathogens after injection.

Ifng enhanced C3 mRNA and protein expression levels in a human astroglioma cell line [37]. Ifng increased steady state mRNA levels of both C3 and C4 in three different human cell types (Hep G2, U937, and primary fibroblasts). Ifng stimulation also increased C3 and C4 protein synthesis [38]. Moreover, Ifng induced biosynthesis of complement components C2, C4, and Cfh at the transcriptional level in human proximal tubular epithelial cells [39]. In mice, Cfb mRNA expression was synergistically upregulated in murine macrophages [40], and Ifng directly stimulated transcription or stabilized complement C3 and C4 mRNAs [41]. Transgenic mice over-expressing Ifng in the brain exhibited neuroinflammation induced by upregulation of the early components of the complement cascade, including C3 and C4a [41]. Thus, increased C3 and C4 mRNA expression following CAWS administration appears to be due to increased Ifng expression, which may occur due to recognition of CAWS as a pathogen.

Interferon regulatory factors, including Irf1 (Figure 5F, Figure 6C), Irf7 (Figure 6C), and Irf9 (Figure 5F), are Ifng-induced genes encoding transcription regulators that induce the expression of other immune-related genes, including genes that mediate the antiviral activity of Ifng and play pivotal roles in the regulation of cellular responses, such as inflammation in host defense [42]. Indeed, Ifng enhanced the expression of Itgam [43], Icam1 [44], and IL-12rb1 [45]. In particular, Irf1, which regulates the expression of Ifn-induced genes and type I interferon, is an early signal that promotes inflammation; Irf1-deficient mice are protected from autoimmune brain inflammation through regulation of Th2-type cytokines [46]. Moreover, expression levels of Irf1 were significantly higher in the synovium of joints with rheumatoid arthritis than in that of those with osteoarthritis [47]. Irf1 gene activation by reactive oxygen species is an early signal that promotes inflammation after ischemic renal injury [48]. Notably, Ifng increases the expression of Irf9 protein in human PBMCs [49], and Cebpb (Figure 6F), which plays a fundamental role in regulating activated macrophage functions, is necessary for Ifng-induced upregulation of Irf9 mRNA in cultured mouse bone marrow-derived macrophages [50]. Cebpb is also involved in expression of Ccl4 mRNA (Figure 6F) induced by Ifng and lipopolysaccharide [51].

The low but similar expression levels of Dectin-2 (Additional file 1: Figure S7) in B6 and DBA/2 mice suggest that Dectin-2 plays similar roles in CAWS-vasculitis in these two mice. The lethality of CAWS-vasculitis in DBA/2 mice, but not in B6 mice, may be due to the increased expression and activity of components in the Ifng-complement axis. Dectin-1, a β-glucan specific receptor, may not be involved in CAWS-vasculitis, because CAWS is a mannoprotein and lacks the dectin-1 ligand, beta-1,3-glucan [52], which results in no signal being transmitted *via* the dectin-1 route. A recent report suggests that the mannan-Dectin-2/FceRIc pathway promotes phosphorylation of a protein called linker for activation of B cells (Lab), which induces natural killer and T cell-mediated Ifng production following *C. albicans* infection [53].

**Figure 7:**
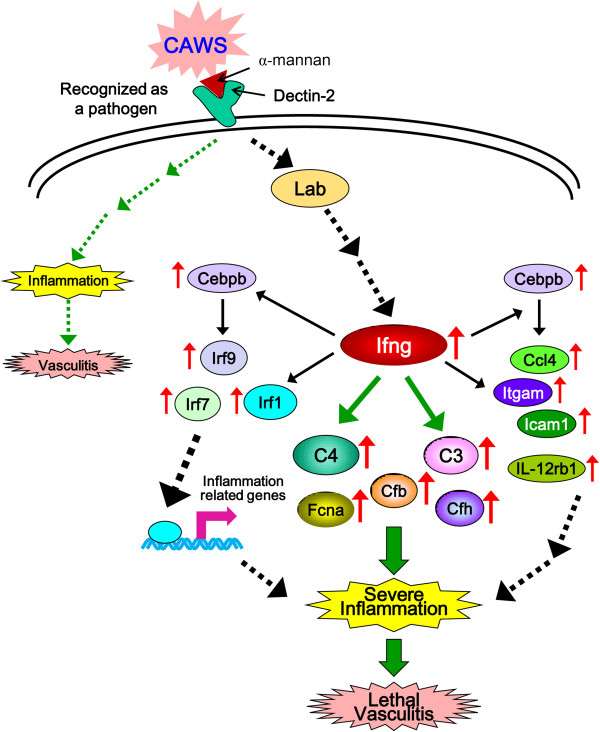
**A molecular model to elucidate the pathogenesis of severe CAWS-vasculitis in DBA/2 mice.** Putative expression targets of Ifng are graphically represented. Upregulated genes following CAWS administration in DBA/2 mice are indicated by upward red arrows. Black and green arrows between the proteins represent known interactions. Dotted arrows indicate indirect interactions. See text for details.

Based on these observations, we propose the following molecular model to explain how CAWS administration causes lethal vasculitis in DBA/2 mice but not in B6 mice (Figure 7). Following injection into the mouse body, the α-mannan component of CAWS binds to the α-mannan receptor Dectin-2 and is recognized as a pathogen, putatively leading to induced Ifng expression. One downstream pathway, shown by dotted green arrows, may lead to mild inflammation and non-lethal vasculitis in both B6 and DBA/2 mice. The other pathway, shown by dotted black arrows, results in enhanced expression of Ifng. Ifng then enhances C3 and C4 (and possibly Cfb, Cfh, and Fcna) expression, which leads to neutrophil activation and causes a cytokine/chemokine storm, inducing damage and necrosis of vascular endothelial cells [13], severe inflammation and finally, a lethal vasculitis. Ifng also enhances expression of Irf1, Irf7, and Irf9, transcription factors that regulate the expression of immune-related genes that may also lead to severe inflammation of the arteries and subsequent lethal vasculitis. Cebpb expression induced by Ifng may play a role in enhanced expression of Ifg9 and Ccl4, which also leads to arterial inflammation, and Itgam, Icam1, and IL-12rb1 induced by Ifng may also participate in inducing severe inflammation.

It was recently shown that CAWS-treated DBA/2 mice developed left ventricular dilution and dysfunction with macroscopic cardiomegaly and concentric left ventricular hypertrophy [13]. We propose here that this chronic mortality of CAWS-treated DBA/2 mice is due to abnormal activation of the complement system induced by Ifng upregulation. This proposal is supported by recent reports showing that C5a elicits anti-inflammatory action through C5L2, and that inflammation in DBA/2 mice deficient in C5 cannot be inhibited following CAWS treatnment [17,20]. The proposal is also supported by the observation that inflammation cannot be inhibited in a mutant mouse deficient in C5 expression with acute pancreatitis and associated lung injury [19]. CAWS also induced vasculitis in other mouse strains, such as C3H/HeN, C3H/HeJ (TLR4 mutation), DBA/1, A/J, CBA/N, C57Bl, AKR, and BALB/c mice [4]. It would be interesting to test whether the Ifng-complement axis is also activated following CAWS administration in these mouse models in the future.

We and others have reported that complement activation is involved in the pathogenesis of systemic autoimmune diseases [14,54-56]. In active anti-neutrophil cytoplasmic antibody-associated vasculitis with renal lesions, significantly higher levels of plasma C3a, C5a, soluble C5b-9, and Bb, a component of the alternative complement pathway, was observed during active development of the disease [54]. Ficolin-1 mRNA level showed a four-fold increase in the PBMCs of TA patients, and that ficolin-1 protein expression was observed in the inflamed regions of surgical aorta specimens, in particular in CD68-positive cells (macrophages or dendritic cells), which are involved in the induction of inflammation [55]. Ficolin-1 mRNA level was also higher in PBMCs isolated from microscopic polyangiitis (MPA) patients than in PBMCs isolated from healthy volunteers [56]. Moreover, increased expression of ficolin-1 protein was also detected by immunostaining in renal specimens taken from MPA patients, and many of the ficolin-1 positive cells coincided with CD68-positive cells in the glomeruli of MPA patients [56]. These results suggest that enhanced expression of ficolin-1 in monocytes and macrophages is also involved in the pathogenesis of human vasculitis.

## Conclusions

Our results suggest that CAWS administration induces non-lethal CAWS-vasculitis in both B6 and DBA/2 mice via a Dectin-2 pathway, but induces enhanced expression of Ifng only in DBA/2 mice, which leads to augmented expression of C3, C4, Cfb, Cfh, and Fcna, thereby causing lethal inflammation of the arteries and severe vasculitis. Thus, the Ifng-complement axis may play an important role in the pathogenesis of lethal CAWS-vasculitis, and suggest that this axis may also be important in the pathogenesis of human vasculitis.

## Methods

### Mouse and fungi

Male B6 and DBA/2 mice were obtained from Japan SLC and Charles River Japan. Mice (5–14 weeks of age) were raised in a specific pathogen-free environment. All animal experiments were performed at the Tokyo University of Pharmacy and Life Sciences (TUPLS), and the experimental protocol was approved by the Committee of Laboratory Animal Experiments of TUPLS. Fungi (*C. albicans* strain NBRC1385) was acquired from the National Institute of Technology and Evaluation Biological Resource Center, stored on Sabouraud agar medium (Difco) at 25°C, and subcultured once every 3 months.

### Preparation of CAWS

CAWS was prepared from *C. albicans* strain NBRC1385 by conventional methods. Culture was performed in 5 liters of C-limiting medium for 2 days at a rotation speed of 400 rpm while pumping in air at 5 L/min and 27°C. After culturing, an equal volume of ethanol was added, and after allowing it to stand undisturbed overnight, the precipitate was recovered. This fraction was dissolved in 250 ml of distilled water, ethanol was added, and the solubilized fraction was again allowed to stand undisturbed overnight. The precipitate was recovered and dried with acetone to obtain CAWS.

### RNA isolation

Total RNA of leukocytes from whole blood of B6 and DBA/2 mice was stabilized by RNAlater™ (Ambion) and then isolated using LeukoLOCK™, a total RNA Isolation System (Ambion, Austin, TX, USA), according to the manufacturer’s instructions. The integrity of the total RNAs used for the microarray analysis was confirmed using the RNA 6000 Nano LabChip Kit (p/n 5067–1511) on the Agilent 2100 Bioanalyzer (G2938C; Agilent Technologies Inc., Palo Alto, CA), and only samples with an RNA integrity number (RIN) above 8.8 were used for gene expression profiling.

### DNA microarray analysis

Microarray analyses were performed as single-color hybridizations. Total RNAs obtained from mouse PBMCs were independently reverse- transcribed using oligo-dT primers containing the T7 RNA polymerase promoter sequence to generate cDNAs and MMLV-RTase. These were then subjected to *in vitro* transcription using T7 RNA polymerase to label the cRNAs with Cy3-CTP (Amersham Pharmacia Biotech, Piscataway, NJ) using a Fluorescent Linear Amplification Kit (Agilent Technologies). Purified Cy3-labeled cRNAs (825 ng) from individual B6 or DBA/2 mice were then hybridized on the microarrays. Washing, scanning, and gene analysis with Agilent’s Whole Mouse Genome Microarray (4×44K; G4122F), on which 31,226 known genes were spotted, were conducted according to the manufacturer’s protocol (Agilent Technologies). Agilent Feature Extraction software (v. 9.5.1) was used to assess spot quality and extract feature intensity statistics. The Subio Platform and Subio Basic Plug-in (v1.11; Subio Inc., Aichi, Japan) were then used to calculate the between-sample fold change. When a large number of genes were automatically classified by the software into a signaling pathway heatmap, only the genes whose mRNA levels were distinct between B6 and DBA/2 mice following CAWS treatment were selected. The details of the microarray data have been deposited in the Gene Expression Omnibus (GEO; http://informahealthcare.com/doi/abs/10.3109/08923973.2013.830124) database (accession number GSE44803).

### Histological examination

Mouse tissues were fixed in 4% paraformaldehyde immediately after removal, then embedded in paraffin and cut into sections (4 μm thick). Sections were stained with H&E according to standard procedures.

### Expression profiling using quantitative reverse transcription-polymerase chain reaction (qPCR)

Assay-on-Demand TaqMan probes with relevant primers were used for qPCR analysis using the ABI PRISM 7900 according to the manufacturer’s instructions (PE Applied Biosystems, Foster City, CA). Total RNA (500 ng) obtained using the PAXgene Blood RNA Kit was reverse-transcribed with the High Capacity cDNA Archive Kit (ABI). PCR consisted of an initial denaturation (95°C, 10 min) followed by 40 cycles of denaturation (95°C, 15 sec) and annealing/extension (60°C, 1 min). A standard curve was generated from the amplification data for each primer using a dilution series of PBMC RNA as the template. Fold change values were normalized to GAPDH expression levels using the standard curve method according to the manufacturer’s protocol.

### Ethical permission

All experiments in which mice were used were performed with the approval of the ethics committee of Tokyo University of Pharmacy and Life Sciences.

## Abbreviations

Asb2: Ankyrin repeat and SOCS box-containing; B6: C57BL/6J; CAWS: *Candida albicans* water-soluble fraction; Cebpb: CCAAT/enhancer-binding protein beta; C3: Complement C3; C3ar1: Component 3a receptor 1; C4: Complement C4; C5: Complement C5; C5ar1: Component 5a receptor 1; C6: Complement C6; C7: Complement C7; C9: Complement C9; Cfb: Complement factor b; Cfh: Complement factor h; Cfi: Complement factor i; Fcna: Ficolin-A; dsRNA: Double-stranded RNA; dsRBD: Double-stranded RNA binding domain; Eif2ak2: Eukaryotic translation initiation factor 2-alpha kinase 2; cRNAs: Complementary RNAs; HC: Hemolytic complement; H&E: Hematoxylin and eosin; HV: Healthy volunteers; Ifng: Interferon gamma; Irf1: Interferon regulatory factor-1; Lat: Linker of activated T cells; Lbp: Lipopolysaccharide-binding protein; Mbl: Mannose-binding lectin; Pact: Protein activator of the interferon-induced protein kinase; PBMC: Peripheral blood mononuclear cell; PBS: Phosphate buffered saline; Pkr: Protein kinase R; qRT-PCR: Quantitative real-time polymerase chain reaction; Trf: T cell-replacing factor

## Competing interests

The authors declare that they have no competing interests.

## Authors’ contributions

NO and HN designed the research and HN wrote the draft of the manuscript. DO performed the DNA microarray analysis. NM, KT, and NO designed and performed the experiments in mice. HN and DO were involved in assessment and interpretation of DNA microarray data. MS, NM, and AI performed pathological analysis. All authors read and approved the final manuscript.

## Supplementary Material

Additional file 1 **Supplementary Results.** Supplementary References. **Figure S1.** Histopathological analysis of aortic roots in B6 and DBA/2 mice at 2 w, 4 w, 8 w, and 9 w following CAWS administration. **Figure S2.** Expression profiles of genes whose mRNA levels were altered following administration of CAWS to B6 and DBA/2 mice. **Figure S3.** Expression profiling of the microarray data for genes involved in cytokine signal transduction pathways following administration of CAWS to B6 and DBA/2 mice. **Figure S4.** Expression profiling of the microarray data for immune-related genes following administration of CAWS to B6 and DBA/2 mice. **Figure S5.** Expression profiling of the microarray data for autophagy-related genes following administration of CAWS to B6 and DBA/2 mice. **Figure S6.** Expression profiling of the microarray data for autophagy-related genes following administration of CAWS to B6 and DBA/2 mice. **Figure S7.** Line graphs and scatter plots for Pkr, Dectin-1, and Dectin-2 mRNA levels. **Table S1.** Upstream regulator analysis of Ifng. Click here for file
