# Supplementary material for: CAWS administration increases the expression of interferon γ and complement factors that lead to severe vasculitis in DBA/2 mice
Source: BMC Immunol. 2013 Sep 24;14:44. doi: 10.1186/1471-2172-14-44 (PMC3876726; doi:10.1186/1471-2172-14-44)
Supplement: Additional file 1 — Supplementary Results. Supplementary References. Figure S1. Histopathological analysis of aortic roots in B6 and DBA/2 mice at 2 w, 4 w, 8 w, and 9 w following CAWS administration. Figure S2. Expression profiles of genes whose mRNA levels were altered following administration of CAWS to B6 and DBA/2 mice. Figure S3. Expression profiling of the microarray data for genes involved in cytokine signal transduction pathways following administration of CAWS to B6 and DBA/2 mice. Figure S4. Expression profiling of the microarray data for immune-related genes following administration of CAWS to B6 and DBA/2 mice. Figure S5. Expression profiling of the microarray data for autophagy-related genes following administration of CAWS to B6 and DBA/2 mice. Figure S6. Expression profiling of the microarray data for autophagy-related genes following administration of CAWS to B6 and DBA/2 mice. Figure S7. Line graphs and scatter plots for Pkr, Dectin-1, and Dectin-2 mRNA levels. Table S1. Upstream regulator analysis of Ifng. [file 1471-2172-14-44-S1.pdf]

SUPPLEMENTARY DATA

Expression of complement factors is induced in DBA/2 mice following administration of *C. albicans* water-soluble mannoprotein-beta-glucan complex (CAWS)

Noriko Nagi-Miura[1, ‡], Daisuke Okuzaki[2, 3, ‡], Kosuke Torigata[2], Minami A. Sakurai[2], Akihiko Ito[4], Naohito Ohno[1, †] and Hiroshi Nojima[2, 3,*]

Supplementary Results

CAWS disturbed cytokine signaling in DBA/2 mice. We surveyed cytokine signaling and found that IL-8 and IL-12 signaling pathways were disturbed in DBA/2 mice injected with CAWS (Fig. S3). In the “IL-8 signaling” pathway (Fig. S3A), two genes were moderately upregulated (black arrows in Fig. S3A): integrin alpha-M (Itgam or CD11b) and intercellular adhesion molecule-1 (Icam1 or CD54). Enhanced expression of Itgam in DBA/2 mice may reflect the activation of leukocytes involved in the innate immune system such as monocytes, granulocytes, macrophages, and natural killer cells, because Itgam is a subunit of the heterodimeric integrin alpha-M beta-2 (αMβ2) molecule expressed on the surface of these leukocytes that mediates inflammation by regulating phagocytosis, cell-mediated cytotoxicity, and chemotaxis[1]. Itgam is also involved in the complement system due to its capacity to bind inactivated complement component 3b (iC3b). Icam-1, a transmembrane protein that belongs to the immunoglobulin superfamily, is an integrin alphaMbeta2 ligand; leukocytes bind to endothelial cells via the ICAM-1/ integrin complex and then transmigrate into tissues[2]. Since Icam-1 is induced by cytokine stimulation, enhanced Icam-1 in DBA/2 mice may be due to augmented cytokine signaling.

In the “IL-12 signaling” (Fig. S3B), “Differential regulation of cytokine production” (Fig. S3C), and “Role of cytokines in mediating communication” (Fig. S3D) pathways, Ifng (red arrow) and seven designated genes (black arrows) were upregulated: interleukin 12 receptor beta 1 (IL-12rb1), interleukin 4 (IL-4), macrophage stimulating 1 (Mst1), Cebpb (see Fig. 5C), Sfpi1 (see Fig. 5D), chemokine (C-C motif) ligand 4 (Ccl4; Fig. S3C) and interleukin 25 (IL-25; Fig. S3D). Peak induction of IL-12rb1, IL-4, and Ccl4 at 4 w suggests that they may play a causative role in the hypersensitive immune response in DBA/2 mice. Because Mst1 and IL-25 were enhanced only at a later time point (9 w), their expression may result from the gene changes that occur at earlier time points observed in DBA/2 mice.

The scatter plot of the genes highlighted by red and black arrows in Figure S3A revealed high mRNA levels in DBA/2 at 2 w or 4 w following CAWS administration, suggesting that changes in their mRNA levels according to the heatmap (Fig. S3E) are physiologically significant (Fig. S3F). By contrast, mRNA levels of Afpi1 and IL-25 are too low to determine their physiological significance.

CAWS induced upregulated expression of immune-related genes only in DBA/2 mice. In the “Toll-like receptor signaling” pathway (Fig. S4A), expression of two genes were conspicuously enhanced in DBA/2: eukaryotic translation initiation factor 2-alpha kinase 2 (Eif2ak2; black arrow) and Lbp (see Fig. 5C; turquoise arrow). Eif2ak2, also known as protein kinase R (Pkr), is activated by virally synthesized double-stranded RNA (dsRNA), protein activator of the interferon-induced protein kinase or heparin, and plays a pro-apoptotic function through its N-terminal dsRNA binding domain (dsRBD) and C-terminal kinase domain. Since Eif2ak2 is induced by interferon, the observed Eif2ak2 induction may result from enhanced expression of Ifng, as described above. Following CAWS treatment, dsRNA components or materials recognized as dsRNA bind to and activate Eif2ak2, which stimulates activation of nuclear factor-kB (NF-kB), a critical transcription factor for inflammation, by phosphorylating its inhibitory subunit (IkB).

In the “Communication between innate and adaptive immune cells” (Fig. S4B), “T cell receptor signaling” (Fig. S4C), and “Altered T cell and B cell signaling in rheumatoid” (Fig. S4D) pathways, Ifng (see Figures 2, 5 and 6) and thee other genesare upregulated (red and black arrows): C-X-C motif chemokine 10 (CXCL10; black arrow in Fig. S4B), IL-4 (see Fig. S3B), and linker of activated T cells (Lat) (Fig. 7D). CXCL10 is secreted in response to IFN-γ and plays a role in chemoattraction for macrophages, T cells, NK cells, and dendritic cells[3]. Because CXCL10 is an interferon gamma-induced protein, its enhanced expression results from augmented expression of interferon gamma (see Figures 2, 5 and S3). Since Lat was enhanced only at the late time point (9 w), its expression may result from the gene changes that occur at earlier time points observed in DBA/2 mice.

Scatter plot of the genes highlighted by arrows in Figure S4A show that the mRNA levels of Lat, Ifng, IL-4, and Eif2ak2 were high enough to conduct physiologically significant comparison of mRNA expression levels according to the heatmap (Fig. S4E) in DBA/2 mice at both 2 w and 4 w following CAWS administration (Fig. S4F). By contrast, Lbp mRNA levels were high only at 4 w and CXCL10 expression was low at both 2 w and 4 w, suggesting that their augmented expression is not physiologically significant.

CAWS induced upregulated expression of autophagy-related genes only in DBA/2 mice. Notably, expression of several autophagy-related genes was upregulated only in DBA/2 mice (highlighted by red font and black arrows in Fig. S5A): Ifng (see Figures 2, 5 and 6), Eif2aka (see Fig. S4A), autophagy-related protein 7 (Atg7), G protein-coupled receptor 182 (Gpr182), and “protein tyrosine phosphatase receptor type, f polypeptide interacting protein alpha 4” (Ppfia4). Atg7 is essential for the induction of autophagic cell death[4]. GPR182 is an orphan G protein-coupled receptor of unknown function, although inhibition of human GPR182 mRNA expression by siRNA has been shown to increase autophagy in H4 cells [5]. Ppfia4, or liprin alpha4, belongs to the liprin-alpha gene family of kinesin-cargo linkers and is a candidate inhibitory drug target for modulation of autophagy in neurodegenerative disease (Lipinski et al., 2010). These results suggest that autophagy is somehow involved in the pathogenesis of CAWS-related symptoms in DBA/2 mice.

Scatter plot analysis of the expression of genes highlighted by arrows in Figure S5A

showed that mRNA levels of Ppfia4, Ifng, and Eif2ak2 are high enough to conduct physiologically significant comparison between B6 and DBA/2 mice based on their heatmaps (Fig. S5B) at both 2 w and 4 w following CAWS administration (Fig. S5C), suggesting that their augmented expression is physiologically significant. By contrast, mRNA levels of Atg7 and Gpr182 were too low to conclude that heatmap comparison of these genes in B6 and DBA/2 may not be physiologically significant.

Ifng is predominantly activated in DBA/2 mice following CAWS administration. Genes (fold change ≥2.0 and P = ≤0.05) that were differentially regulated in B6 and DBA/2 mice following CAWS administration were uploaded into the Ingenuity Pathway analysis program (Ingenuity H Systems, www.ingenuity.com) for upstream regulator analysis (see Supplementary Table 1S for details). This program is useful for defining biological interactions predicted to be upregulated or downregulated based on the expression changes observed in the gene sets. Statistical analysis of these predictions are carried out for calculation of the activation z-score, which is designed to ensure that gene sets composed of randomly chosen perturbed genes with random fold changes do not produce significant results on average according to the Ingenuity Downstream Effect Analysis whitepaper. The results indicated that the predicted activation state of Ifng is shown as activated only in B6 mice within 8 w following CAWS administration. On the other hand, they showed that Ifng was activated in DBA/2 mice within 4 w, 8 w and 9 w following CAWS administration. As expected, we found that a greater activation z-score of Ifing was predicted in DBA/2 mice following CAWS administration.

Expression levels of Pkr and Pkr-related genes. Levels of Pkr were also examined because it is produced in large amounts in response to signals from cells infected by cytolytic viruses and functions to phosphorylate and inactivate the translation factor eIF-2, thereby reducing protein synthesis within the cell to inhibit viral propagation and induce apoptosis. Indeed, mRNA levels of Pkr were also induced in DBA/2 mice but not in B6 mice (red lines in Fig. S4A), although less significantly than those of Ifng. mRNA levels of some Pkr-related genes were also augmented following CAWS administration (Fig. 5B). Scatter plot analysis indicated that although the mRNA level for Pkr is not high, it is sufficient for concluding that its heatmap between B6 and DBA/2 is significantly different (Fig. S4B).

Detailed explanation of the supplementary figures

Figure S1. Histopathological analysis of aortic roots in B6 and DBA/2 mice at 2 w, 4 w, 8 w, and 9 w following CAWS administration. Control mice (no CAWS) were sacrificed at 2 w following intraperitoneal injection of PBS for 5 consecutive days. Tissue sections were stained with H&E and observed under a microscope. Bar = 200 µm.

Figure S2. Expression profiles of genes whose mRNA levels were altered following administration of CAWS to B6 and DBA/2 mice. (A) Line graphs showing the mRNA levels detected by 41,000 probes and the log2 ratio of the normalized processed signal following administration of CAWS into B6 and DBA/2 mice. The color bar on the right

side of the panel indicates the log2 ratio for each mRNA at 2 w, 4 w, 8 w, or 9 w following CAWS administration versus that at 0 w. Intensity gradients indicate the relative mRNA level: blue (down-regulation) corresponds to a log2 ratio of -2, and crimson (upregulation) corresponds to a log2 ratio of 2. (B) The mosaic tiles for the entire set of microarray data are presented after hierarchical clustering based on Pearson's correlation. Tile colors indicate the mean relative mRNA levels at 2 w, 4 w, 8 w, or 9 w following CAWS administration versus 0 w; blue tiles correspond to a log2 ratio of -5 (down-regulation), red tiles correspond to a log2 ratio of 5 (upregulation), and gray tiles indicate no change (see the rightmost color bar). The turquoise arrow indicates the notable cluster of genes including C3 and C4, in which red tiles are observed only in DBA/2 mice.

Figure S3. Expression profiling of the microarray data for genes involved in cytokine signal transduction pathways following administration of CAWS to B6 and DBA/2 mice. Mosaic tiles and hierarchical clustering of microarray data are shown for the genes involved in IL-8 signaling (A), IL-12 signaling and production in macrophages (B), differential regulation of cytokine production (C), and the role of cytokines in mediating communication between immune cells (D). B6 and DBA/2 samples were clustered using a hierarchical clustering program (Spearman) to identify gene-to-gene relationships. Ifng is highlighted by red arrows, while Itgam, Icam1, IL-12rb1, IL-4, Mst1, Cebpb, Sfpi1, Ccl4, and IL-25 are highlighted by black arrows. (E) Intensity gradients indicate the mean value of the expression level (log2 ratio): blue (down-regulation) and crimson (upregulation) are shown compared to the average value at 0 w after CAWS administration (gray). (F) Scatter plots of highlighted genes in the log of signal intensity at 2 w (left panel) or 4w (right panel) versus 0 w following CAWS administration in DBA/2 mice; data are plotted along the vertical and horizontal axes (arbitrary unit: a.u.), respectively. Ifng is highlighted by red font.

Figure S4. Expression profiling of the microarray data for immune-related genes following administration of CAWS to B6 and DBA/2 mice. Mosaic tiles and hierarchical clustering of microarray data are shown for genes involved in the Toll-like receptor signaling pathway (A), communication between innate and adaptive cells (B), T cell receptor signaling (C), and altered T cell and B cell signaling in rheumatoid (D). B6 and DBA/2 samples were clustered using a hierarchical clustering program (Spearman) to identify gene-to-gene relationships. Eif2ak2, Lat, Cxcl10, and IL-4 genes are highlighted by black arrows, while Ifng and Lbp genes are highlighted by red and turquoise arrows, respectively. (E) Intensity gradients indicate the mean value of the expression level (log2 ratio): blue (down-regulation) and crimson (upregulation) are shown compared to the average value at 0 w after CAWS administration (gray). (F) Scatter plots of highlighted genes in the log of signal intensity at 2 w (left panel) or 4 w (right panel) versus 0 w following CAWS administration in DBA/2 mice; data are plotted along the vertical and horizontal axes (arbitrary unit: a.u.), respectively.

Figure S5. Expression profiling of the microarray data for autophagy-related genes following administration of CAWS to B6 and DBA/2 mice. (A) Mosaic tiles and hierarchical clustering of microarray data are shown. B6 and DBA/2 samples were

clustered using a hierarchical clustering program (Spearman) to identify gene-to-gene relationships. Atg7, Eif2ak2, Gpr182, and Ppfia4 are highlighted by black arrows, while Ifng is highlighted by a red arrow. (B) Intensity gradients indicate the mean value of the expression level (log2 ratio): blue (down-regulation) and crimson (upregulation) are shown compared to the average value at 0 w after CAWS administration (gray). (C) Scatter plots of highlighted genes in the log of signal intensity at 2 w (left panel) or 4 w (right panel) versus 0 w following CAWS administration in DBA/2 mice; data are plotted along the vertical and horizontal axes (arbitrary unit: a.u.), respectively.

Figure S6. Expression profiles of interferon genes following administration of CAWS to B6 and DBA/2 mice. Mosaic tiles and hierarchical clustering of microarray data are shown. B6 and DBA/2 samples were clustered using a hierarchical clustering program (Spearman) to identify gene-to-gene relationships. Colors with intensity gradients indicate the mean value of the expression level (log2 ratio): blue (down-regulation) and crimson (upregulation) are shown in comparison with the average value at 0 w (gray).

Figure S7. Line graphs and scatter plots for Pkr, Dectin-1, and Dectin-2 mRNA levels. (A) Line graphs showing the mRNA levels of Pkr (red), Dectin-1 (green) and Dectin-2 (blue) following administration of CAWS to B6 and DBA/2 mice. (B) Scatter plots for Pkr, Dectin-1, and Dectin-2 mRNA levels in the log of signal intensity at 2 w (left panel) or 4 w (right panel) versus 0 w following CAWS administration in DBA/2 mice; data are plotted along the vertical and horizontal axes (arbitrary unit: a.u.), respectively.

Supplementary References

1. Solovjov DA, Pluskota E, Plow EF: **Distinct roles for the alpha and beta subunits in the functions of integrin alphaMbeta2.** *J Biol Chem* 2005, **280**, 1336-1345.
2. Yang L, Froio RM, Sciuto TE, Dvorak AM, Alon R, Luscinskas FW: **ICAM-1 regulates neutrophil adhesion and transcellular migration of TNF-alpha-activated vascular endothelium under flow.** *Blood* 2005, **106**, 584-592.
3. Dufour JH, Dziejman M, Liu MT, Leung JH, Lane TE, Luster AD: I**FN-gamma-inducible protein 10 (IP-10; CXCL10)-deficient mice reveal a role for IP-10 in effector T cell generation and trafficking.** *J Immunol* 2002, **168**, 3195-3204.
4. Shen S, Kepp O, Kroemer G: **The end of autophagic cell death?** *Autophagy* 2012, **8**, 1-3.
5. Lipinski MM, Zheng B, Lu T, Yan Z, Py BF, Ng A, Xavier RJ, Li C, Yankner BA, Scherzer CR, Yuan J: **Genome-wide analysis reveals mechanisms modulating autophagy in normal brain aging and in Alzheimer's disease.** *Proc Natl Acad Sci USA* 2010, **107**, 14164-14169.

Table S1. Upstream regulator analysis of Ifng.

**Analysis**	**Gene name**	**Predicted Activation State**	**Activation z-score**	**p-value of overlap**	**Gene regulation consistent with activation or inhibition (Number of genes regulated of all potential gene targets)**
B6 4 weeks (365)	IFNG		**-0.862**	3.16E-03	***AGT***, ***ALDH1L1***, ***ALOX15***, *ARG1,ASS1,C1QA,C1QC,C1R,C3,C4B,CFB,CP,* ***Cyp2c40***, ***CYP2E1***, *FBP1,FN1,* ***FOS***, *GJB2,* ***IGF1***, ***IRG1***, ***LCN2***, *LIFR,* ***Retnla***, ***SAA2***, *SERPINA1,SERPING1,* ***ZFP36*** *(27 of 29)*
B6 8 weeks (241)	IFNG	**Activated**	**3.284**	2.00E-11	***ABCA6***, *ARG2,* ***BST1***, ***CASP4***, *CCNO,* ***CLEC4E***, *CLEC5A,FAM123A,* ***FGL2***, ***FPR2***, ***HCAR2,ID1***, ***IFITM1***, ***IFITM2,IL1B***, ***IL1RN***, ***IL36G***, ***IRG1***, ***Klra17***, ***LCN2***, ***MEFV***, *MMP9,* ***MT1E***, *PIGR,* ***S100A8***, ***S100A9***, *SAA2,* ***SIRPA***, *SLC40A1,* ***SOCS3***, *TREM1,TREM3,* ***Wfdc17***, ***Wfdc18***, ***ZFP36*** *(35 of 38)*
B6 9 weeks (969)	IFNG		**0.735**	2.67E-02	***ABCA6***, ***AGT***, ***ALDH1L1***, *ARG1,ARG2,ASS1,* ***ATF3***, ***BATF2***, *C1R,CAPN3,* ***CD80***, ***CFTR***, *CLEC5A,CP, Cxcl12,* ***Cyp2c40***, ***CYP2E1***, *FBP1,* ***FHL2***, ***FPR2***, ***GJA5***, *GJB2,* ***ICOSLG***, ***IFITM1***, ***IGF1***, ***IL1B***, ***IL1RL1,IRG1***, ***LAMC2***, ***LCN2***, ***LIX1***, *MYOD1,PIGR,* ***POU4F2***, *PPP1R1B,SAA2,SERPINA1,SERPING1,SLC9A3, TGFBR3,TGM1,TIMP4,TREM1,Wfdc17,Wfdc18 (45 of 55)*
DBA/2 4 weeks (139)	IFNG	**Activated**	**3.705**	6.89E-23	***AIF1***, *ALOX15,* ***ARG1***, *ARG2,* ***ATF3***, ***BST1***, ***C1QA***, ***C1QC***, ***C1R,C4B***, ***CCL3L1/CCL3L3***, ***CCL4***, *CCNO,* ***CD14***, *CD163,* ***CFB***, ***CLEC4E***, ***CTSL2***, *EDNRB,* ***FCGR1A***, ***FN1***, ***FOS***, ***HCAR2***, ***HSPA1A/HSPA1B***, ***IER3***, ***IL1B,IL36G***, ***IRG1***, ***Klra17***, ***MEFV,*** *MST1R,* ***OAS1***, ***OAS2***, ***OASL***, *Retnla,* ***RTP4***, *SAA2,* ***SLFN5***, *SLPI,TREM1,* ***Wfdc17*** *(41 of 42)*
DBA/2 8 weeks (376)	IFNG	**Activated**	**5.612**	4.95E-25	***AIF1***, ***ARG1***, *ARG2,* ***ATF3,BATF2***, ***BST1,C3***, ***CASP4***, *CCNO,* ***CCRL2***, ***CD14***, ***CFB***, ***CLEC4E***, *CLEC5A,* ***CXCL10***, ***CXCL9***, *EHF,FAM123A,* ***FAS,FCGR1A***, ***FCGR2A***, ***FCGR3A***, ***FPR2***, ***Gbp2***, ***GBP2***, ***GBP4***, ***IER3***, ***Ifi202b***, ***Ifi204***, ***IFIH1***, ***IFITM1,IFITM2***, ***IL18BP***, ***IL1B***, ***IL1RN***, ***IL36G***, ***IRF7,IRG1***, ***IRGM***, ***Klra17***, ***LCN2***, ***Ly6a***, ***MEFV***, *MMP9,* ***NAMPT***, ***OAS1***, ***OAS2***, ***OASL***, *Retnla,* ***RTP4***, ***S100A8***, ***S100A9***, *SAA2,* ***SELP***, ***Serpina3g***, *SLC40A1,* ***SLFN5***, *SLPI,* ***TLR4***, ***TNFRSF1A***, *TREM1,TREM3,TYROBP,* ***USP18***, ***Wfdc17***, ***Wfdc18*** *(66 of 69)*
DBA/2 9 weeks (180)	IFNG	**Activated**	**3.661**	1.54E-08	***AIF1***, ***ATF3***, *CAMK4, CCNO,* ***CCRL2***, ***CD14***, ***CFB***, ***CLEC4E***, ***CXCL10***, ***FCGR1A***, ***FOS***, ***HSPA1A/HSPA1B***, ***IER3***, ***IL1B***, ***IRG1***, ***KCNJ1***, ***LCN2***, ***MEFV***, ***OAS2***, *SAA2,* ***SELP***, ***SOCS3***, *STAT6,* ***TMOD1***, ***Wfdc17***, ***Wfdc18*** *(26 of 27)*

Gene names in Bold indicate increased expression after CAWS administration, normal italic font indicates decreased expression.

Supplementary Figures

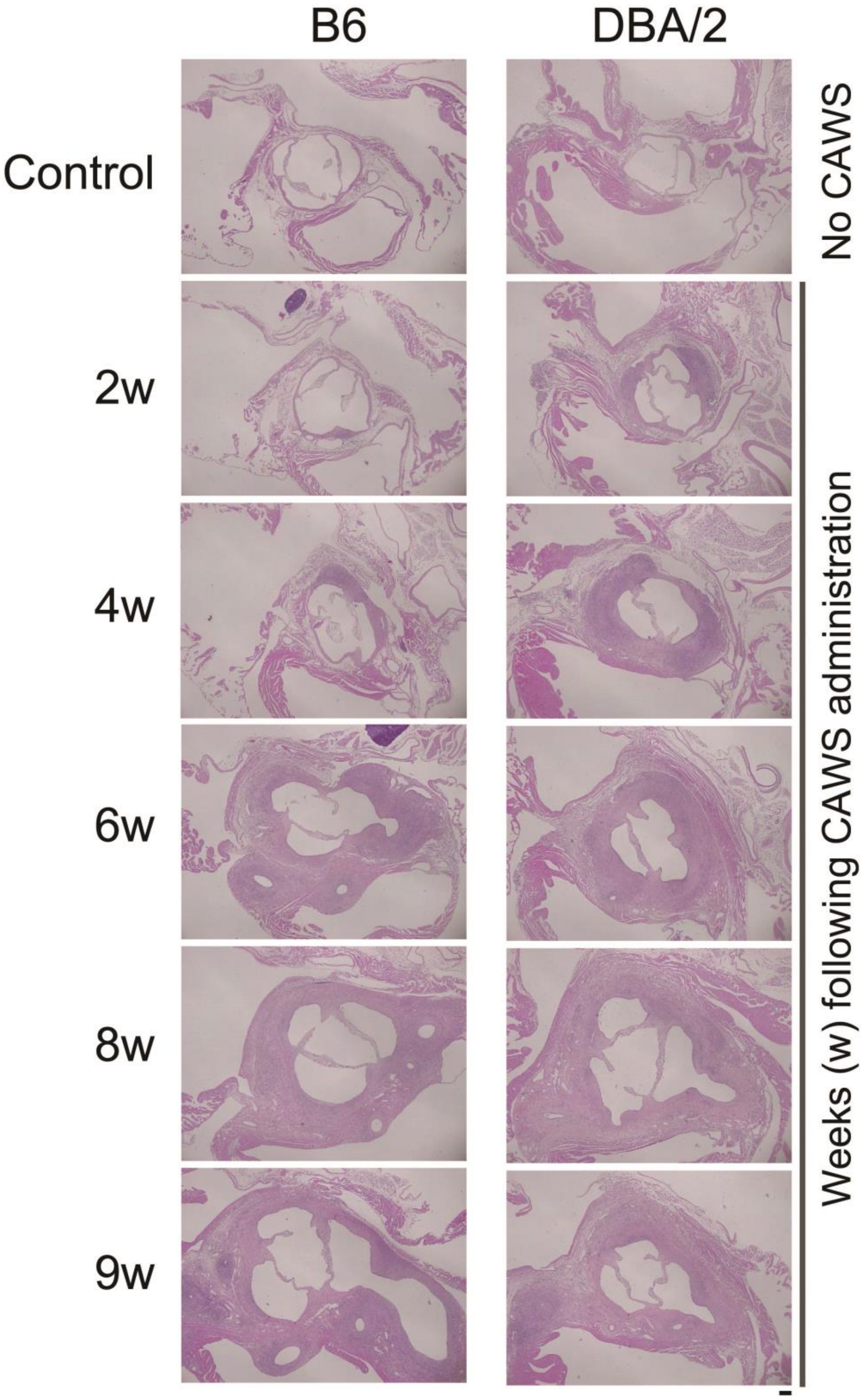

Figure S1. Histopathological analysis of aortic roots in B6 and DBA/2 mice at 2 w, 4 w, 8 w, and 9 w following CAWS administration.

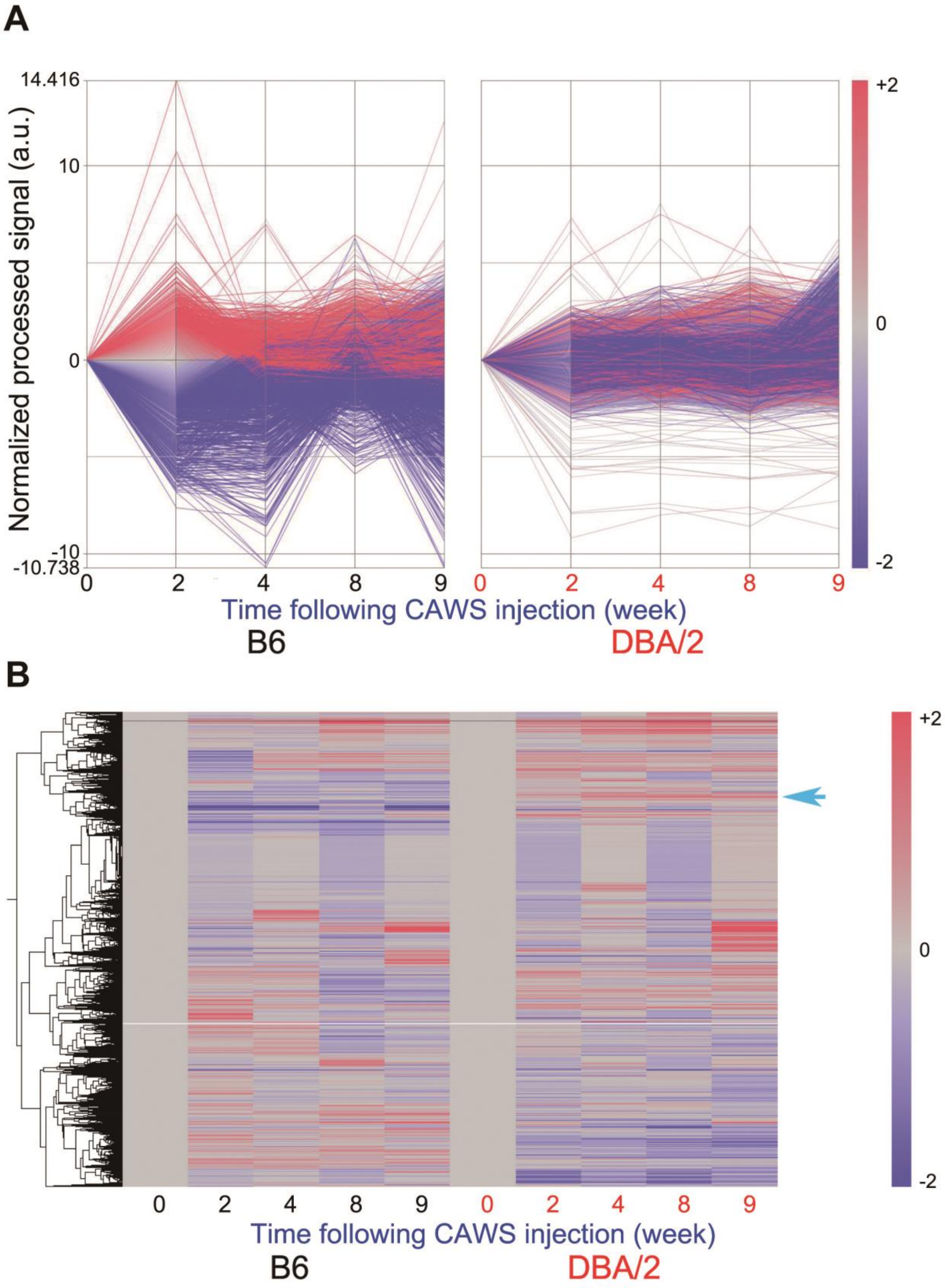

Figure S2. Expression profiles of genes whose mRNA levels were altered following administration of CAWS to B6 and DBA/2 mice.

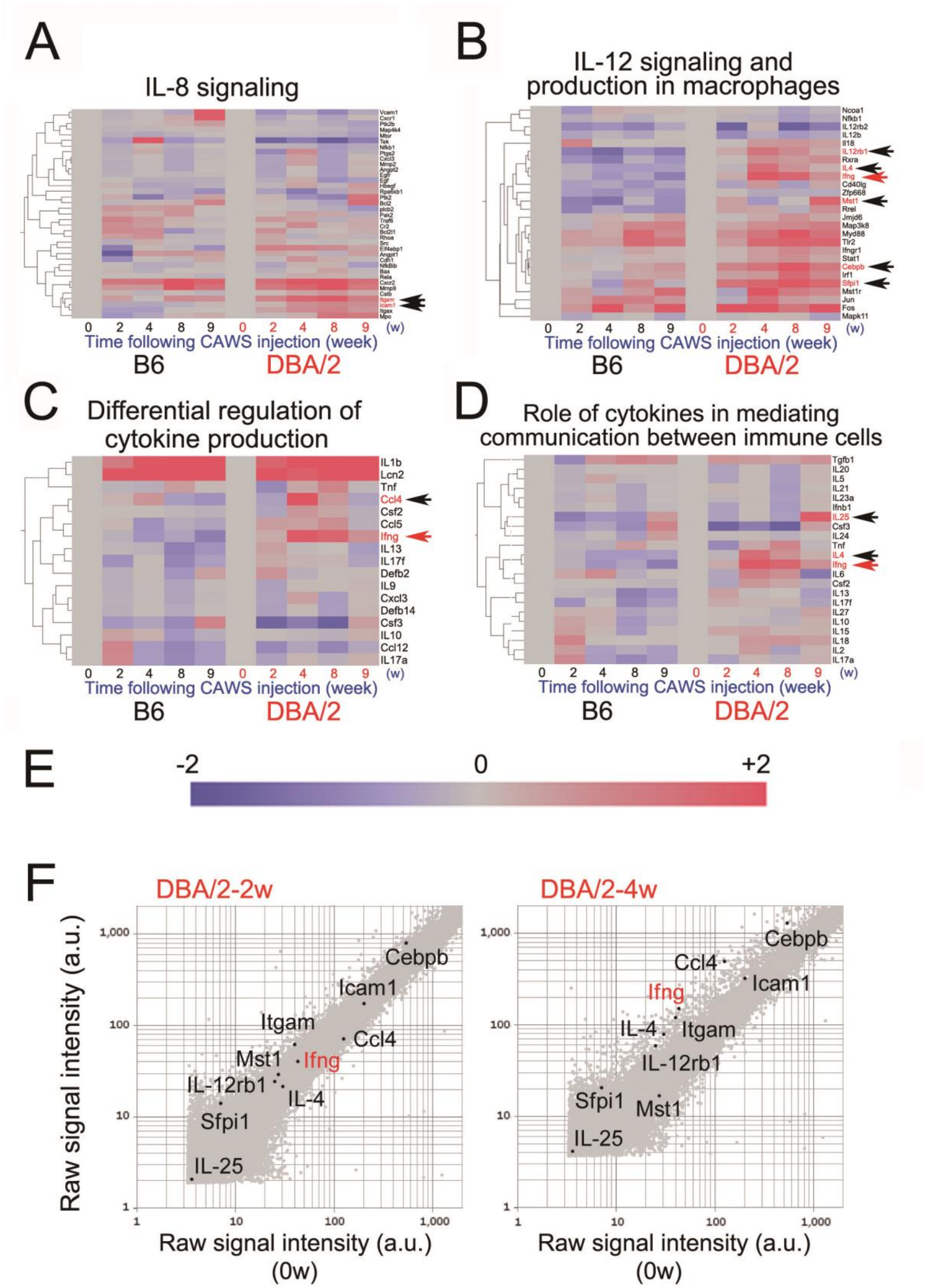

Figure S3. Expression profiling of the microarray data for genes involved in cytokine signal transduction pathways following administration of CAWS to B6 and DBA/2 mice.

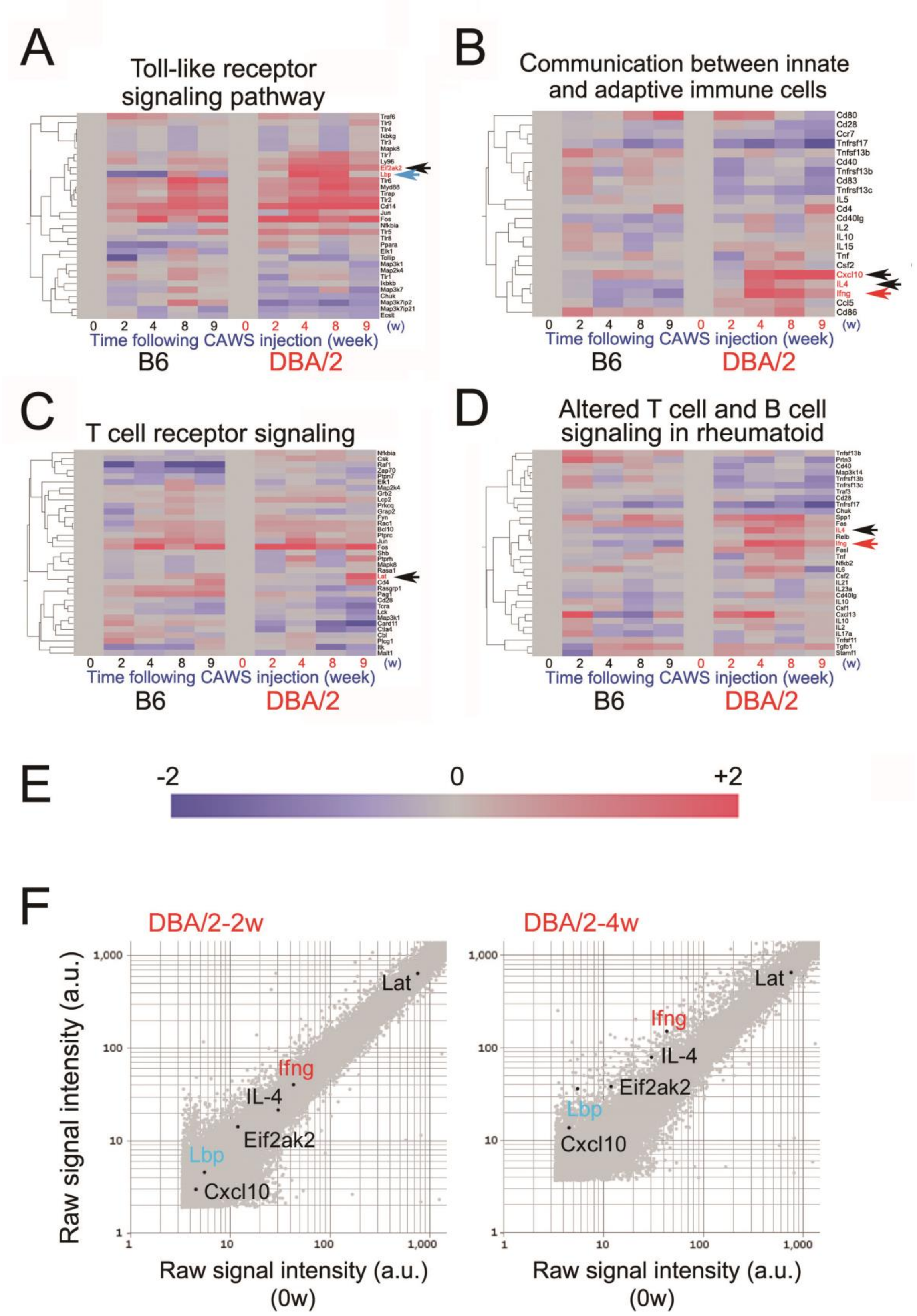

Figure S4. Expression profiling of the microarray data for immune-related genes following administration of CAWS to B6 and DBA/2 mice.

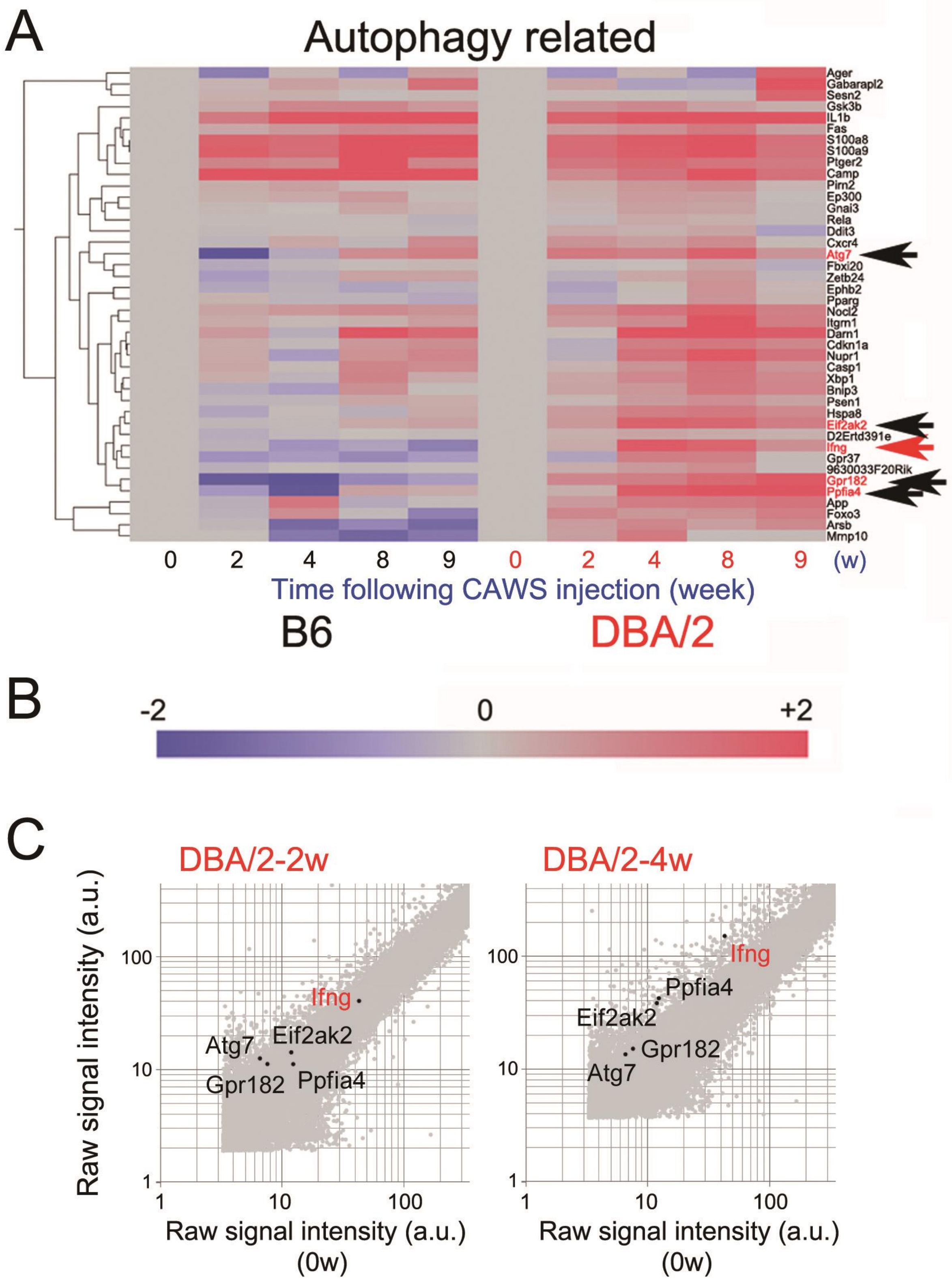

Figure S5. Expression profiling of the microarray data for autophagy-related genes following administration of CAWS to B6 and DBA/2 mice.

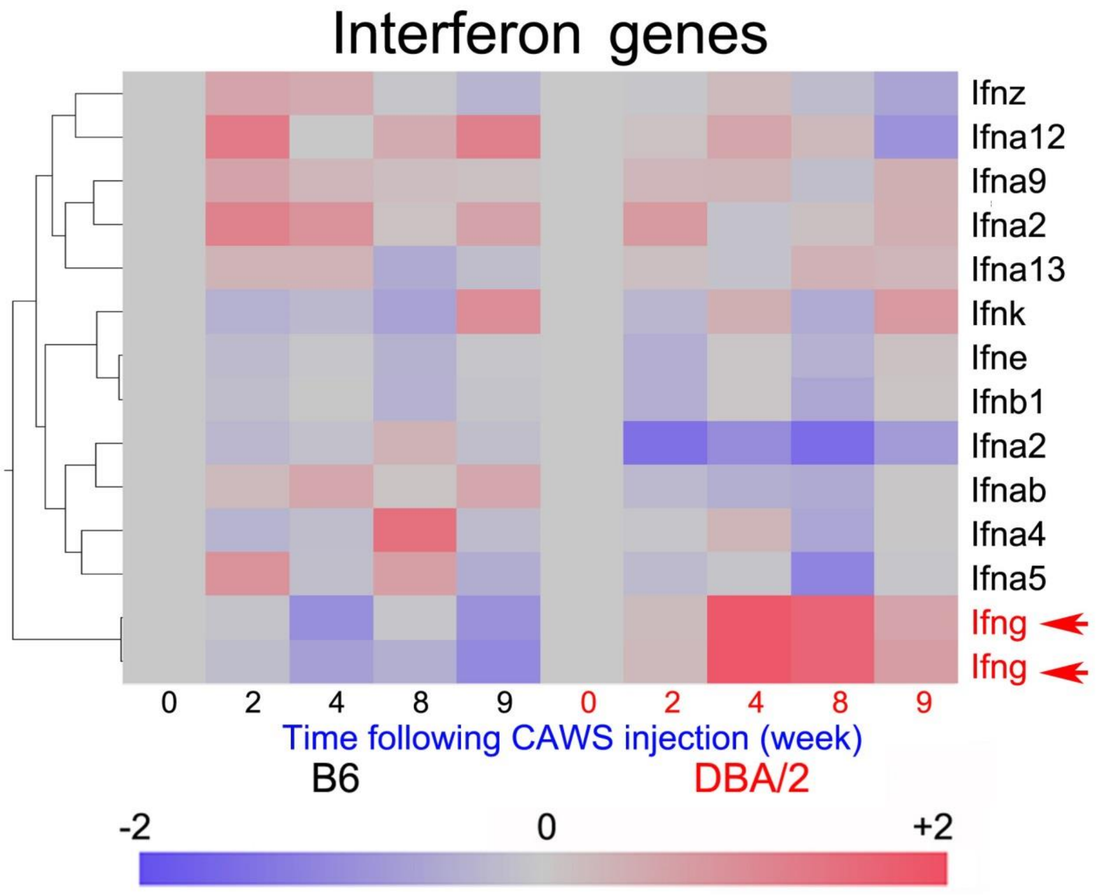

Figure S6. Expression profiles of interferon genes following administration of CAWS to B6 and DBA/2 mice.

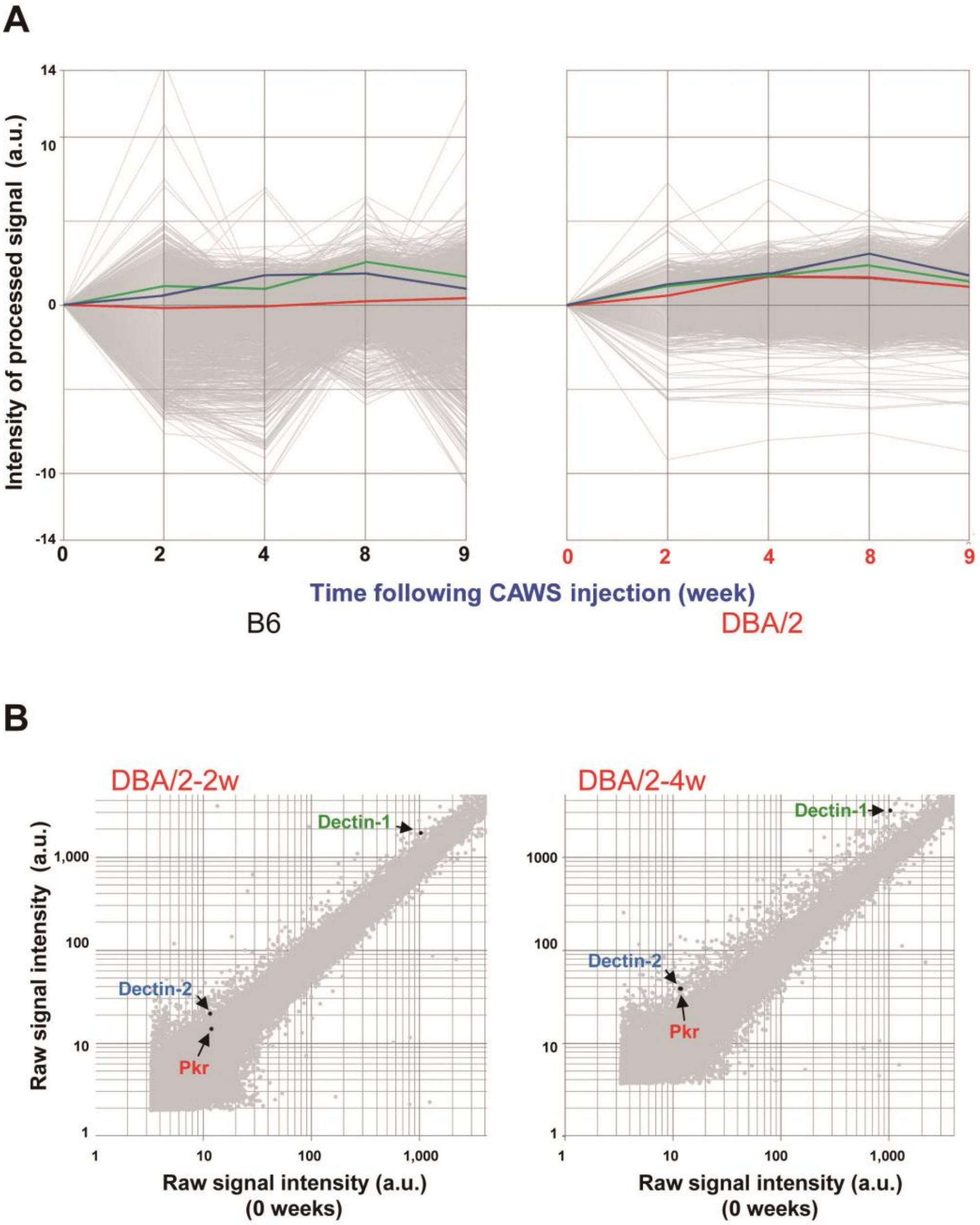

Figure S7. Line graphs and scatter plots for Pkr, Dectin-1, and Dectin-2 mRNA levels.
